# At neutral pH the chronological lifespan of *Hansenula polymorpha* increases upon enhancing the carbon source concentrations

**DOI:** 10.15698/mic2014.06.149

**Published:** 2014-05-20

**Authors:** Adam Kawałek, Ida J. van der Klei

**Affiliations:** 1 Molecular Cell Biology, Groningen Biomolecular Sciences and Biotechnology Institute, Systems Biology Centre for Metabolism and Ageing, University of Groningen, the Netherlands.

**Keywords:** Hansenula polymorpha, chronological lifespan, ageing, acidification, dietary restriction

## Abstract

Dietary restriction is generally assumed to increase the lifespan in most eukaryotes, including the simple model organism *Saccharomyces cerevisiae*. However, recent data questioned whether this phenomenon is indeed true for yeast. We studied the effect of reduction of the carbon source concentration on the chronological lifespan of the yeast *Hansenula polymorpha* using four different carbon sources. Our data indicate that reduction of the carbon source concentration has a negative (glucose, ethanol, methanol) or positive (glycerol) effect on the chronological lifespan. We show that the actual effect of carbon source concentrations largely depends on extracellular factor(s). We provide evidence that *H. polymorpha* acidifies the medium and that a low pH of the medium alone is sufficient to significantly decrease the chronological lifespan. However, glucose-grown cells are less sensitive to low pH compared to glycerol-grown cells, explaining why only the reduction of the glycerol-concentration (which leads to less medium acidification) has a positive effect on the chronological lifespan. Instead, the positive effect of enhancing the glucose concentrations is much larger than the negative effect of the medium acidification at these conditions, explaining the increased lifespan with increasing glucose concentrations. Importantly, at neutral pH, the chronological lifespan also decreases with a reduction in glycerol concentrations. We show that for glycerol cultures this effect is related to acidification independent changes in the composition of the spent medium. Altogether, our data indicate that in *H. polymorpha* at neutral pH the chronological lifespan invariably extends upon increasing the carbon source concentration.

## INTRODUCTION

Dietary restriction (DR) is defined as reduction in nutrient availability without malnutrition 1,2. DR has been proposed to be a general intervention to prevent ageing in a large variety of organisms, ranging from simple model organisms like yeast to higher eukaryotes, such as rodents 3,4,5.

The reason as to why DR enhances yeast lifespan is currently debated. The chronological lifespan (CLS) of yeast is defined as the time non-dividing cells remain viable after exit from the growth phase 6,7. In *Saccharomyces cerevisiae* DR is typically defined as the reduction of the glucose concentration in the batch medium from 2% to 0.5%. *S. cerevisiae* is a Crabtree-positive yeast, meaning that mitochondrial oxidative metabolism is repressed in media with high concentrations of glucose. In 2% glucose containing media, the carbon source is initially fermented to ethanol, which is subsequently utilized when glucose is depleted. This results in the so called diauxic shift 8. During glucose fermentation *S. cerevisiae* also secretes acetate. Is has been argued that acetate secretion together with the acidification of the medium to values below pH 4.5 is the major cause of the reduced lifespan of *S. cerevisiae* cells grown on 2% glucose 8,9,10. Indeed the pH of *S. cerevisiae* cultures containing synthetic complete medium supplemented with 2% glucose can drop to values of 2.5-2.8 9,11, whereas cultures containing 0.5% glucose do not acidify 12. Also, buffering the medium to pH 6.0 or resuspension of stationary cells in water has been shown to extend the CLS of yeast cells grown on media containing 2% glucose 9.

So far, the effects of DR, acidification and acetate on yeast CLS were mainly studied using *S. cerevisiae *and glucose as a carbon source. Here we study the effects of these parameters using the Crabtree negative yeast *Hansenula polymorpha*. This yeast is unable to inhibit respiration in the presence of high levels of glucose in favour of fermentation and is generally assumed not to secrete acetate during growth on glucose. In addition to the effect of different glucose concentrations on the CLS, we studied the effects of different carbon source concentrations and acidification when cells were grown on alternative carbon sources, namely glycerol, ethanol and methanol. Our data indicate that reduction of the carbon source concentration can have a negative (glucose, ethanol, methanol) or positive (glycerol) effect on the CLS of *H. polymorpha*. However, at neutral pH the CLS invariably increases upon enhancing the carbon source concentration. This indicates that reducing the carbon source concentration is not a common intervention to enhance yeast lifespan. Also, we show that a low pH especially reduces the CLS of *H. polymorpha* after exit from the growth phase and acts independent of the presence of compounds (like acetate) secreted in the medium during the growth phase.

## RESULTS

### The effect of carbon source concentration on the chronological lifespan is carbon source dependent

Recent reports indicated that medium composition strongly influences yeast CLS 2,11,13. The type of nutrient limitation as well as the concentration of amino acids required for auxotrophic laboratory strains strongly affect the survival of the cells in the stationary phase 14,15,16. To avoid these issues we performed our studies using a prototrophic *H. polymorpha* strain and mineral media (MM) in which the carbon source is the only limiting factor for growth (meaning that the cells exit the growth phase solely due to depletion of the carbon source). We therefore defined DR in our current study as a reduction in carbon source concentration under conditions that all other medium components are present in excess. At these conditions a reduction in carbon source concentration results in a proportional reduction in the growth yield (final optical density). We used methylamine as N-source in all our studies, as we recently showed that this results in a longer CLS of *H. polymorpha* relative to the use of ammonium sulphate 17,18.

**Figure 1:**
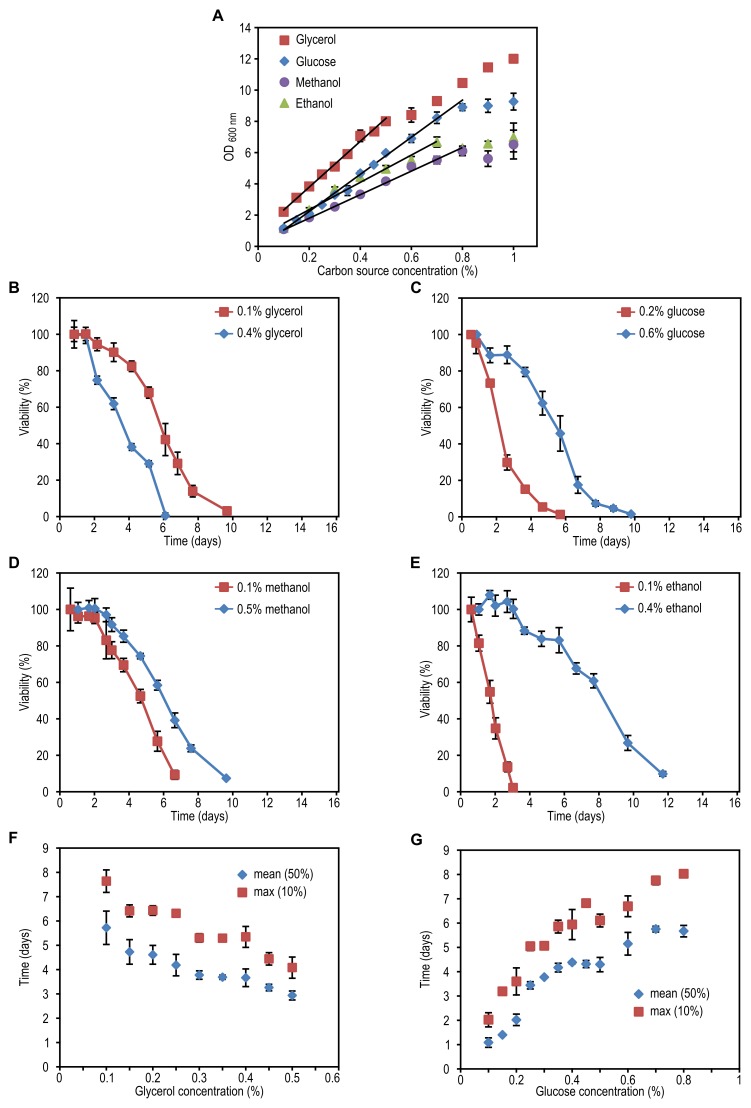
FIGURE 1: The effect of carbon source concentration on yeast chronological lifespan. *H. polymorpha* cells were grown on various concentrations of the indicated carbon sources and 0.25% methylamine as nitrogen source. The OD_600 nm_ was measured when cultures did exit the growth phase **(A)**. Lines indicate ranges in which a linear correlation (R>0.98) between final OD and carbon source concentration was observed. Data represent mean ± SD (n=3). Chronological lifespan of cells grown at a low and a high concentration of glycerol **(B)**, glucose **(C)**, methanol **(D)**, ethanol **(E)**. Mean lifespan (mean) and maximum lifespan (max) were calculated as the time when cultures reach 50% and 10% viability, respectively, for cultures grown on different concentrations of glycerol **(F)** or glucose **(G)**. Data represent mean ± SD from 4 to 12 independent cultures.

To determine suitable low and high carbon source concentrations for the four different carbon sources used in this study (glycerol, glucose, methanol, ethanol), we grew wild-type (WT) *H. polymorpha* strain at various concentrations of these compounds and determined the optical densities (OD) of the stationary cultures. A linear increase in final OD with increasing carbon source concentrations was observed up to 0.5% glycerol, 0.8% glucose, 0.8% methanol and 0.7% ethanol (Fig. 1A). Based on these data we selected the low concentrations as those that resulted in final ODs between 1 and 2, whereas for the high concentrations we chose those that resulted in ODs of 1 - 2 OD units below the maximal OD obtained with the highest concentrations of the linear range (Fig. 1A).

The lifespan of cells grown on the high glycerol concentration was shorter compared to cells grown on a low concentration of glycerol (Fig. 1B). However, when glucose, methanol or ethanol was used, the opposite was observed, namely an increase in CLS with increasing carbon source concentration (Fig. 1C-E).

To further analyse the observed opposite effects of reducing the carbon source concentrations on CLS for different carbon sources, we confined our further studies using glycerol, as an example of a carbon source for which reduction in the concentration enhanced the lifespan, and glucose, as representative of a carbon source for which the opposite was observed.

**Figure 2:**
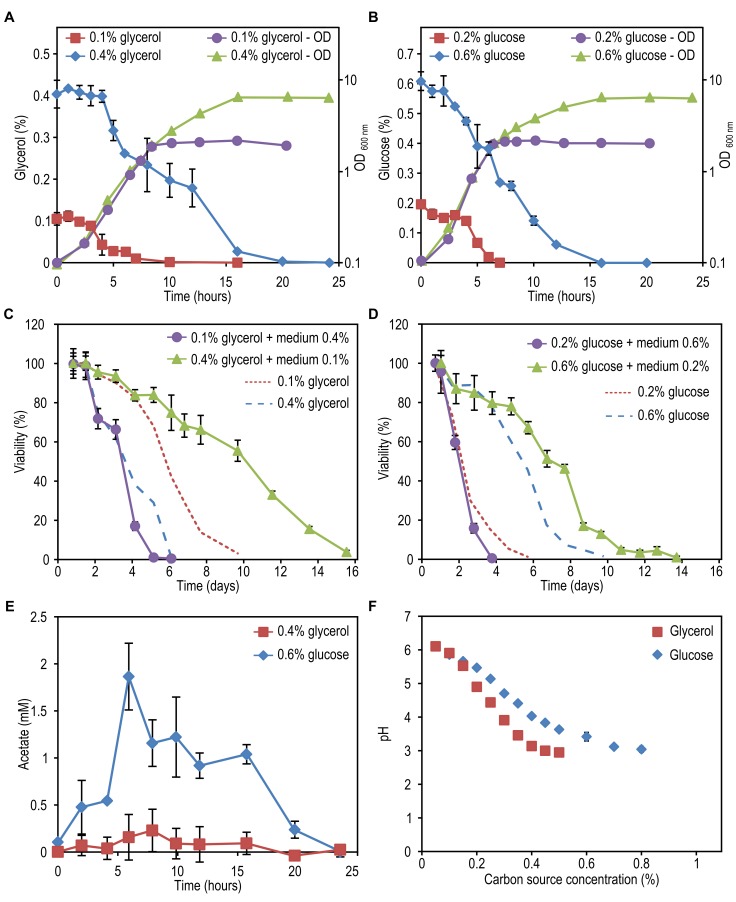
FIGURE 2: The impact of extracellular factors on DR mediated lifespan changes. Cells were grown on 0.1% and 0.4% glycerol **(A)** as well as 0.2% and 0.6% glucose **(B)**. Growth and carbon source depletion were monitored in time. Data represent mean glycerol or glucose concentration ± SD (n=3) and mean OD_600nm_ ± SD. Cells were grown on 0.1% and 0.4% glycerol **(C)** or 0.2% and 0.6% glucose **(D)**. Spent medium of cultures grown on one concentration of carbon source was replaced by the spent medium originating from cultures grown on the second concentration of the same carbon source and viability of the cultures was measured in time. Data represent mean viability ± SD from 3 independent cultures. Lines indicating viability of cultures left in its own medium were redrawn from Figure 1B and 1C. **(E) **The concentration of acetate was measured in clarified medium at different time points upon shifting the cells to medium containing 0.4% glycerol or 0.6% glucose and 0.25% methylamine. Data represent mean ± SD (n=3). **(F)** Cells were grown on different concentrations of glycerol or glucose. The pH of the cultures was measured at the beginning of the stationary phase. Data represent mean ± SD (n=3).

Analysis of the residual glucose and glycerol concentrations confirmed that indeed at the chosen concentrations for these two carbon sources, the cultures did exit the growth phase due to carbon source depletion (Fig. 2A-B). At maximum concentration analysed for glucose, readdition of glucose to the spent medium allowed growth of the cells, however, the doubling time and final OD were not as high as for fresh mineral medium (Fig. S1). Moreover when higher concentrations, namely 1 or 2% of glycerol or glucose, were used, these carbon sources were not depleted when growth ceased (Fig. S2A-B) and hence cells did exit the growth phase because of other, yet unknown, reasons.

Systematic analysis of the CLS of glycerol and glucose cultures using the previously defined ranges of carbon source concentrations revealed that the CLS gradually decreased when the glycerol concentrations increased from 0.1% to 0.5% (Fig. 1F, Table 1). Using higher concentrations of glycerol (1 or 2%) prolonged the lifespan in comparison to 0.5% glycerol (Fig. S2C). The CLS of glucose grown cells gradually increased with increase of glucose concentrations in the range of 0.2 - 0.8% (Fig. 1G, Table 1). The lifespan of the cells grown on 1% or 2% glucose was comparable to the lifespan of cells grown on 0.5% glucose (Fig. S2D).

**Table 1:** Mean and maximum lifespan of *H. polymorpha* WT cells upon growth on different concentrations of glycerol and glucose. The mean lifespan was calculated as a time point when cultures reached 50% viability; the maximum lifespan was calculated as a time point when cultures reached 10% viability. The data represents mean ± SD from at least 4 independent cultures. ND - not determined.

	**Glycerol**		**Glucose**	
**Concentration (%)**	**Mean lifespan (days)**	**Max. lifespan (days)**	**Mean lifespan (days)**	**Max. lifespan (days)**
0.1	5.72 ± 0.69	7.64 ± 0.46	1.08 ± 0.20	2.02 ± 0.29
0.15	4.73 ± 0.51	6.42 ± 0.25	1.40 ± 0.03	3.19 ± 0.03
0.2	4.61 ± 0.39	6.43 ± 0.20	2.02 ± 0.28	3.60 ± 0.60
0.25	4.19 ± 0.44	6.31 ± 0.03	3.44 ± 0.15	5.04 ± 0.18
0.3	3.78 ± 0.17	5.31 ± 0.17	3.77 ± 0.03	5.06 ± 0.03
0.35	3.69 ± 0.09	5.29 ± 0.06	4.17 ± 0.18	5.85 ± 0.27
0.4	3.67 ± 0.36	5.35 ± 0.43	4.38 ± 0.06	5.94 ± 0.62
0.45	3.26 ± 0.13	4.44 ± 0.25	4.31 ± 0.15	6.81 ± 0.15
0.5	2.94 ± 0.18	4.08 ± 0.44	4.29 ± 0.29	6.10 ± 0.27
0.6	ND	ND	5.15 ± 0.51	7.9 ± 0.47
0.7	ND	ND	5.75 ± 0.12	7.75 ± 0.18
0.8	ND	ND	5.67 ± 0.24	8.02 ± 0.15

To measure the viability in CLS experiments, we routinely determine the colony forming units (CFU) using YPD plates. To exclude that the use of glucose plates for both glucose and glycerol cultures affected the results, we also determined the viability using YP-glycerol plates. Plating on YPD and YP-glycerol plates resulted in similar amount of colonies (Fig. S3A). Also very similar CLS curves were obtained when YPD or YP-glycerol plates were used (Fig. S3B-C). Based on this observation we continued our studies using YPD plates.

### Extracellular factor(s) are involved in carbon source concentration-dependent lifespan changes

To test whether the composition of the spent medium differentially affects the CLS in glycerol and glucose media, we performed spent medium swap experiments. Because glycerol and glucose were completely depleted after 20h of growth (Fig. 2A-B), this time point was used to swap the media.

Incubating cells grown on 0.1% glycerol in spent medium of cultures grown on 0.4% glycerol strongly reduced their lifespan to values observed for cultures grown on 0.4% glycerol and kept in this medium (Fig. 2C, Table 2). However, upon incubation of cells grown on 0.2% glucose in spent medium of cultures containing 0.6% glucose, there was no major reduction in lifespan relative to cells grown on 0.2% glucose and kept in their spent medium (Fig. 2D).

**Table 2:** Mean and maximum lifespan of *H. polymorpha* cells grown on low and high concentrations of glycerol and glucose and placed in the spent medium from cultures containing other concentration of same carbon source.

	**Concentration used for growth of cells (%)**	**Concentration used to obtain spent medium (%)**	**Mean lifespan (days)**	**Max lifespan (days)**
Glycerol	0.1	0.4	3.46 ± 0.06	4.58 ± 0.59
	0.4	0.1	10.15 ± 0.23	14.76 ± 0.52
Glucose	0.2	0.6	2.02 ± 0.09	3.08 ± 0.12
	0.6	0.2	7.08 ± 0.59	10.21 ± 0.29

Placing cells grown on 0.4% glycerol into spent medium of cells grown on 0.1% glycerol strongly extended their CLS (Fig. 2C). The mean and maximum lifespan of these cells increased almost three times compared to cells grown on 0.4% glycerol and kept in their spent medium (Table 2 and Table 1). Incubating cells grown in 0.6% glucose in spent medium from cultures grown on 0.2% glucose also extended their lifespan, but the difference was smaller (less than 1.5 fold increase) (Fig. 2D, Table 2 and Table 1).

This data indicates that composition of the spent medium strongly affect the chronological lifespan both for glycerol and glucose containing cultures. Furthermore, the CLS of glycerol-grown cells changed much more (either positively or negatively) upon the medium swap relative to the glucose-grown cells.

### Acetic acid is not a major factor reducing *H. polymorpha* CLS

Despite the fact that *H. polymorpha* is a Crabtree negative yeast, it has been reported that this yeast may secrete acetic acid as a consequence of the overflow metabolism 19. Being a weak organic acid, acetic acid was shown to directly reduce viability of chronologically ageing *S. cerevisiae* cells 9. As shown in Fig. 2E, external acetic acid concentrations up to 2 mM were detected during the growth phase of 0.6% glucose containing cultures, but this compound was subsequently depleted from the medium within 24h (i.e. the beginning of the CLS measurements). When cells were grown on 0.4% glycerol, no significant amounts of external acetic acid were detected. These data indicate that the acetic acid, which is secreted during growth, is not a toxic compound in the spent medium of high glucose or glycerol cultures, that affects the CLS as it is either not present (glycerol) or quickly depleted from the spent medium (glucose).

Medium acidification was previously shown to affect the lifespan of *S. cerevisiae* at a variety of growth conditions 9,11,20. As shown in Fig. 2F, the pH of the cultures grown on 0.4% glycerol or 0.6% glucose significantly decreased to 3.1 and 3.4, respectively, whereas the pH of cultures grown at low carbon source concentrations remained above 5.0. Hence, the reduced CLS of cells grown on low glycerol or glucose media upon incubation in spent medium of high glycerol or glucose cultures could (partially) be explained by the low pH of these solutions. However, it does not explain why the reduction of the CLS of the glycerol-grown cells is much stronger compared to the glucose-grown cells. Possibly, other compounds than acetic acid are present in the medium and toxic at low pH in glycerol-grown cultures, but not or less in glucose cultures. We therefore set out to experiments to further dissect the role of low pH and putative secreted toxic compounds on *H. polymorpha* CLS.

### Low pH of the milieu shortens the chronological lifespan

**Figure 3:**
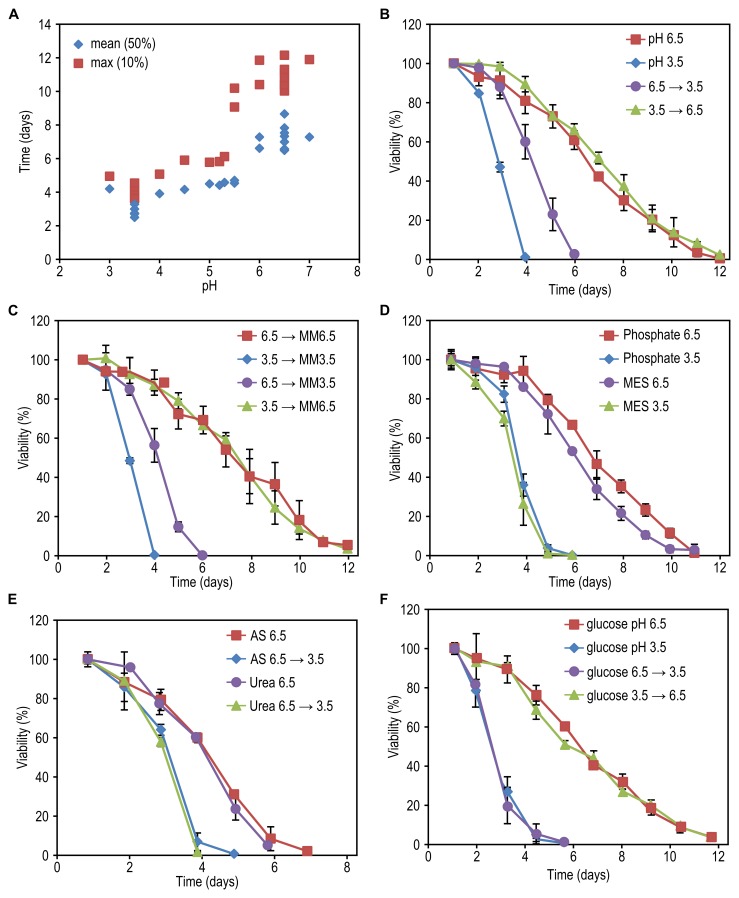
FIGURE 3: The effect of medium pH on yeast chronological lifespan. **(A)** Cells were grown on 0.2% glycerol and 0.25% methylamine in batch fermenters at different pH values. After 20h cells were shifted to flasks and the viability of the cultures was measured over time. Data represent time when viability of the cells reached 50% (mean) and 10% (max) for all individual fermenters. **(B)** Cells were grown on 0.2% glycerol / 0.25% methylamine at pH 6.5 or 3.5. After 20h of cultivation in batch fermenters, the pH of the cultures was changed by the addition of 1 M H_3_PO_4_ (6.5 --> 3.5) or 1 M NaOH (3.5 --> 6.5). Cells before the pH change were kept as the controls. Data represent mean ± SD (n=3). **(C)** Cells were grown on 0.2% glycerol / 0.25% methylamine at pH 6.5 or 3.5. After 20h, spent medium of the cultures was replaced by fresh medium (without carbon source) with a pH of 6.5 (MM6.5) or 3.5 (MM3.5). Data represent mean ± SD (n=3). **(D)** Cells were grown on 0.2% glycerol / 0.25% methylamine at pH 6.5 or 3.5. After 20h, spent medium of the cultures was replaced by potassium phosphate buffer pH 6.5 (phosphate 6.5), pH 3.5 (phosphate 3.5) or MES buffer pH 6.5 (MES 6.5) or pH 3.5 (MES 3.5). Data represent mean ± SD (n=3). **(E)** Cells were grown on 0.2% glycerol and 0.25% ammonium sulphate (AS) or 0.2% urea at pH 6.5. After 20h of cultivation in batch fermenters, the pH of the cultures was changed by addition of concentrated H_3_PO_4_ (6.5 --> 3.5). Cells before the pH change were kept as the controls. Data represent mean ± SD (n=2). **(F)** Cells were grown on 0.4% glucose / 0.25% methylamine at pH 6.5 (glucose pH 6.5) or 3.5 (glucose pH 3.5) in batch fermenters. After 20h the pH of the cultures was changed by addition of H_3_PO_4_ (glucose 6.5 --> 3.5) or NaOH (glucose 3.5 --> 6.5). Cells before the pH change were kept as the controls. Data represent mean viability ± SD (n=2).

For a further detailed analysis of the effect of pH on CLS, we first confined our studies to glycerol containing cultures and one (intermediate) carbon source concentration (0.2%). Cells were grown at different, constant pH values using pH controlled batch fermenters. CLS measurements revealed a sudden increase in mean and maximum lifespan when cells were grown at pH values equal or higher than 6.0, relative to lower pH values (Fig. 3A). These differences are not related to differences in growth rates, because in media with pH values in the range of pH 3.5 to 6.5 the growth curves (doubling time, final OD) were similar (data not shown). Hence, these data confirm that a low pH reduces the CLS of glycerol-grown *H. polymorpha*.

To investigate whether a low pH affects the CLS during the growth, after the growth phase or both, we performed pH swap experiments. Cells were grown on 0.2% glycerol at a constant pH of 6.5 or 3.5 and upon exit from the growth phase, the pH was reduced or increased by the addition of H_3_PO_4_ or NaOH, respectively. Neutralizing the medium of cells grown at pH 3.5 to 6.5 resulted in a similar CLS compared to cells grown and kept at pH 6.5 during the stationary phase (Fig. 3B). Lowering the pH of cultures grown at pH 6.5 to 3.5 resulted in a strong decrease in CLS (Fig. 3B), but the CLS was not shortened to the values observed for cells that were grown and subsequently kept at pH 3.5. These data indicate that a low pH after exit from the growth phase strongly reduces the CLS of glycerol containing cultures.

Next, we asked whether only the low pH is responsible for lifespan shortening or whether it reduced the CLS in combination with other factors in the spent media. For instance, the toxicity of weak organic acids depends on the concentration of the acid and increases with decreasing pH 9,21. Cells were grown in a batch fermenter at a constant pH of 6.5 or 3.5 on 0.2% glycerol. After exit from the growth phase cells were collected by centrifugation and resuspended in fresh mineral medium without carbon source (MM) at a pH of 3.5 or 6.5. Shifting the cells grown at pH 3.5 and pH 6.5 to fresh MM with the same pH did not alter the lifespan in comparison to cells left in spent medium (Fig. 3C, compare with Fig. 3B). Resuspension of the cells grown at pH 3.5 in fresh MM with a pH of 6.5 prolonged the CLS to the same extent as cells grown at pH 6.5 and shifted to mineral medium pH 6.5. Conversely, resuspension of cells grown at pH 6.5 in MM with a pH value of 3.5 shortened the CLS to a similar extent as the pH swap from pH 6.5 to 3.5 in spent medium (Fig. 3C, compare with Fig. 3B).

These data indicate that for cells grown on 0.2% glycerol, a low pH after the growth phase is the major factor reducing the CLS and not toxic components present in the spent medium. This conclusion is furthermore supported by the outcome of experiments in which non-growing cells were transferred to 25 mM phosphate buffer or 50 mM MES buffer with a pH of 3.5 or 6.5 instead of MM (Fig. 3D). Also, the same effect was observed when methylamine was replaced by other nitrogen sources (ammonium sulphate (AS) or urea) (Fig. 3E), indicating that the effect was not specific for cells utilizing methylamine. Importantly, the observed negative effect of a low pH after the growth phase was also observed when cells were grown on 0.4% glucose (Fig. 3F). Hence, the CLS of both glycerol and glucose-grown cells decreases when cells are incubated at a low pH after exit from the growth phase.

### Acidification-independent effect of carbon source concentration on lifespan

**Figure 4:**
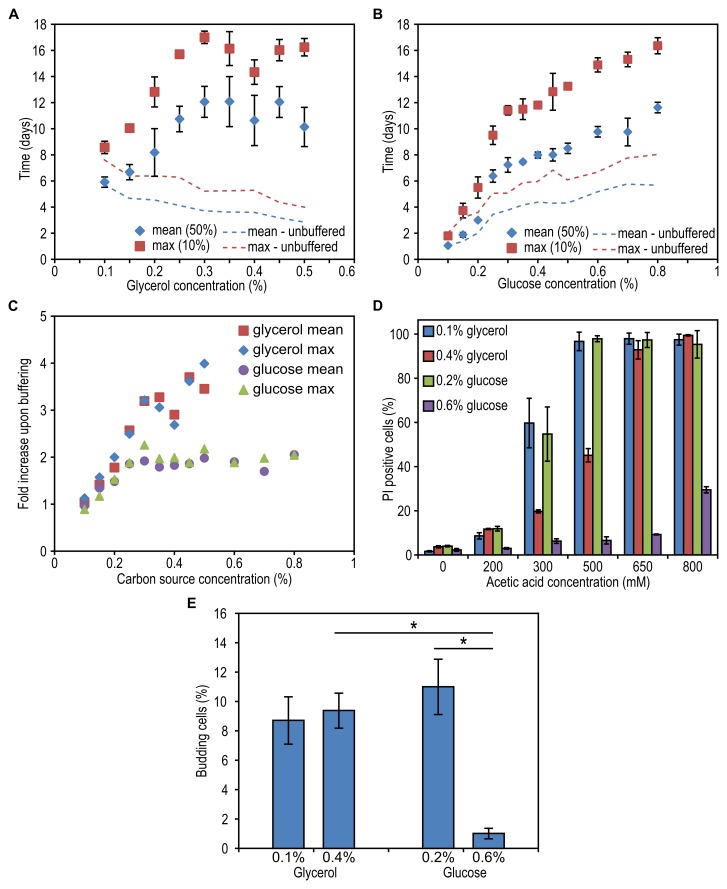
FIGURE 4: The effect of carbon source concentration on yeast lifespan upon buffering of spent medium. Cells were grown on different concentrations of glycerol **(A)** or glucose **(B)** and 0.25% methylamine as the nitrogen source. The pH of the cultures was adjusted to pH 6.5. Data represent mean ± SD from 4 to 8 independent cultures. Trend lines indicating changes in mean and maximum lifespan of cells in non-buffered media were redrawn from Figure 1F and 1G. **(C)** Fold increase of mean and maximum lifespan upon buffering the cultures grown on different concentrations of glycerol and glucose calculated as lifespan after buffering divided by lifespan before buffering.** (D) **The resistance of cells to acetic acid treatment. Upon exit of the growth phase, cells were treated with increasing concentration of acetic acid followed by PI staining and FACS analysis. Data represent mean number of PI positive cells ± SD from 4 cultures. **(E)** The number of budding cells upon exit from the growth phase in unbuffered medium. Data represent mean percentage of cells containing a bud from 6 cultures ± SD.

Although a low pH affects the CLS of both glucose and glycerol-grown cells and a similar acidification is observed for both carbon sources (Fig. 2F), it remained unclear why DR has a positive effect when glycerol is the carbon source and a negative effect when glucose is present in the medium. One explanation may be that the glycerol-grown cells are more sensitive to a low pH. To investigate this, we analysed the effect of neutralizing the pH after exit from the growth phase. Cells were grown in media containing 0.1% to 0.5% glycerol or 0.1% to 0.8% glucose and the pH of the cultures was adjusted to 6.5 when cells stopped to grow. Neutralizing the pH significantly increased the CLS of cells grown on 0.15 to 0.5% glycerol (Fig. 4A, compare with Fig. 1F, Table 1 and 3). Notably, under these conditions, the CLS increased with increasing glycerol concentrations. Neutralizing the medium of glucose-grown cultures also extended the CLS, which increased with increasing carbon source concentrations, like in the non-neutralized cultures (Fig. 4B, compare with Fig. 1G, Table 1 and 3). The CLS extending effect of neutralizing the pH was much less pronounced in glucose-grown cultures in comparison to glycerol-grown cultures (Fig. 4C).

**Table 3:** Mean and maximum lifespan of *H. polymorpha* cells upon growth on different concentrations of glycerol and glucose in medium buffered to pH 6.5. The mean lifespan was calculated as a time point when cultures reached 50% viability; the maximum lifespan was calculated as a time point when cultures reached 10% viability. The data represents mean ± SD from at least 4 independent cultures. ND - not determined.

	**Glycerol**		**Glucose**	
**Concentration (%)**	**Mean lifespan (days)**	**Max. lifespan (days)**	**Mean lifespan (days)**	**Max. lifespan (days)**
0.1	5.92 ± 0.48	8.57 ± 0.40	1.04 ± 0.06	1.79 ± 0.06
0.15	6.68 ± 0.29	10.06 ± 0.59	1.88 ± 0.18	3.73 ± 0.56
0.2	8.19 ± 1.15	12.82 ± 1.83	2.99 ± 0.17	5.50 ± 0.90
0.25	10.75 ± 0.15	15.71 ± 0.98	6.38 ± 0.47	9.50 ± 0.71
0.3	12.06 ± 0.47	17.01 ± 1.19	7.23 ± 0.56	11.42 ± 0.35
0.35	12.08 ± 1.30	16.15 ± 1.92	7.46 ± 0.12	11.49 ± 0.69
0.4	10.64 ± 0.94	14.35 ± 1.92	7.99 ± 0.23	11.81 ± 0.23
0.45	12.06 ± 0.82	16.03 ± 1.18	8.00 ± 0.47	12.83 ± 1.41
0.5	10.14 ± 0.67	16.25 ± 1.50	8.50 ± 0.40	13.25 ± 0.25
0.6	ND	ND	9.77 ± 0.49	14.85 ± 0.63
0.7	ND	ND	9.75 ± 1.06	15.31 ± 0.56
0.8	ND	ND	11.63 ± 0.40	16.36 ± 0.61

We next asked whether the glucose-grown cells are only more resistant to low pH or also against acetic acid. As shown in Fig. 4D, cells grown on 0.6% glucose are also more resistant to short exposure to acetic acid than cells grown on 0.4% glycerol (Fig. 4D) suggesting that an adaptation may have occurred during the growth phase in cultures containing 0.6% glucose.

Exposure to weak organic acids at sublethal concentrations triggers cell cycle exit and prolonged cell stasis rather than cell death. Notably, an efficient cell cycle arrest at G_0_/G_1_ is crucial for longevity of chronologically ageing *S. cerevisiae* 20,22,23,24. To assess the efficiency of cell cycle arrest we counted the percentage of cells in cultures grown on a low and a high concentration of glycerol and glucose after exit from the growth phase. Cultures grown on 0.6% glucose showed almost complete lack of budding cells, whereas more than 8% of the cells grown on 0.2% glucose and 0.1% or 0.4% glycerol contained buds (Fig. 4E).

The above data indicate that buffering has a weaker lifespan extending effect on cells grown on a high concentration of glucose. This effect is accompanied by elevated resistance to acetic acid and a more efficient cell cycle arrest.

### Acidification is not the only extracellular factor in spent medium that affects the chronological lifespan

High carbon source concentrations (glucose or glycerol) are positive for the CLS of *H. polymorpha* cultures when the pH is neutral in the stationary phase. This could be either related to a direct positive effect of the high carbon source levels on the viability of the cells or due to an altered composition of the spent medium (the secretion of higher amounts of compounds that stimulate longevity or depletion of medium component negatively affecting the viability of the cells).

To investigate the acidification-independent effect of spent medium composition we performed buffered spent medium swap experiments. Cells were grown in media containing low or high carbon source concentrations (glycerol or glucose), collected by centrifugation and resuspended in spent medium from cultures containing either the low or the high concentration of carbon source. The pH was adjusted to 6.5 at the onset of the CLS experiment.

**Figure 5:**
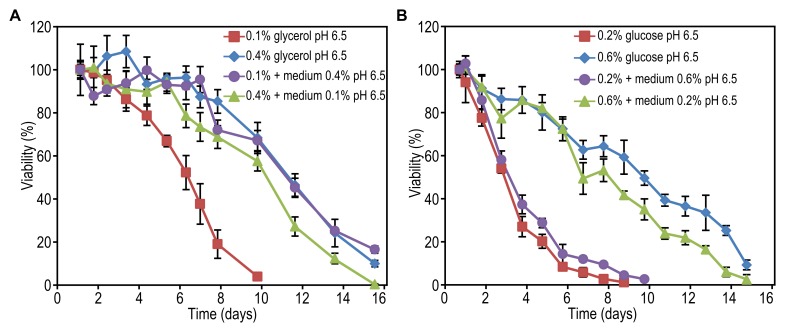
FIGURE 5: Acidification-independent impact of spent medium composition on lifespan. Cells were grown on 0.1% and 0.4% glycerol **(A)** as well as 0.2% and 0.6% glucose **(B)** for 20 hours. The spent medium from cultures grown on one concentration of carbon source was replaced by the spent medium originating from cultures containing other concentration of that carbon source with pH adjusted to 6.5. Data represent mean viability ± SD from 3 independent cultures. Representative data of two experiments with similar results are presented.

Replacing the medium of cells grown on 0.1% glycerol with buffered spent medium from cultures containing 0.4% glycerol strongly extended the CLS to values obtained for cells grown in 0.4% glycerol and buffered upon exit from the growth phase (Fig. 5A, Table 4). Conversely, replacing the medium of cells grown in 0.4% glycerol with buffered medium originating from cultures containing 0.1% glycerol only had a slight reducing effect on the CLS (Fig. 5A). This observation suggests that the spent medium from cultures grown on a low concentration of glycerol possibly contains a factor negatively affecting the lifespan and/or that the spent medium of cells grown on high glycerol contains a factor positively affecting the CLS.

**Table 4:** Mean and maximum lifespan of *H. polymorpha* WT cells grown on low and high concentrations of glycerol and glucose and placed in the buffered spent medium from cultures containing other concentration of same carbon source.

	**Concentration used for growth of cells (%)**	**Concentration used to obtain spent medium (%)**	**Mean lifespan (days)**	**Max lifespan (days)**
Glycerol	0.1	0.4	11.40 ± 0.35	16.04 ± 0.56
	0.4	0.1	10.00 ± 0.42	13.28 ± 0.46
Glucose	0.2	0.6	3.15 ± 0.15	6.67 ± 0.59
	0.6	0.2	7.25 ± 0.94	12.56 ± 0.50

Essentially, similar experiments performed with cultures containing glucose as a carbon source, indicate that in this case extracellular factors only slightly affect the CLS. Replacing the medium with buffered spent medium of cells grown at higher or lower glucose concentrations did not strongly affect the mean and maximum lifespan values (Fig. 5B and Table 4). Based on these observations we conclude that the increased CLS with enhancing carbon source concentration is in part related to the composition of the medium in case of glycerol, but not for glucose.

## DISCUSSION

Dietary restriction is the gold standard intervention that increases the lifespan of many organisms. Reduction of nutrient availability also affects the lifespan of *S. cerevisiae*. The relatively short lifespan and the possibility to use media with various nutrient compositions favour this organism as a model to unravel the mechanism of DR. A disadvantage however is that *S. cerevisiae* can ferment part of the glucose to acetic acid, which, together with the low pH of the spent medium is thought to be the primary cause of life span reduction in 2% glucose containing cultures. The exact mechanisms remain however controversial 9,10,11,12.

Here we analysed the effect of carbon source concentration on the chronological lifespan of the Crabtree negative yeast *H. polymorpha*. Because the effect of glucose concentration on CLS of *S. cerevisiae* was shown to largely depend on nutrient balance and amino acid concentrations in the medium 2, we used in our study mineral media in which the carbon source is the only growth limiting medium compound as well as a prototrophic strain.

Our data indicate that the reduction of carbon source concentration has a negative effect on the CLS when glucose is used, whereas a positive effect was observed for glycerol. Reduction of glucose concentration was previously also shown to drastically reduce the lifespan of another Crabtree negative yeast, namely *Kluyveromyces lactis *25. Spent medium swap experiments revealed that for glycerol, and to the lesser extent for glucose, the effect of changing the carbon source concentrations is dependent on extracellular factors.

Our data indicate that unlike for glucose-grown *S. cerevisiae* 9, acetic acid secretion is not a major factor in CLS reduction for glucose-grown *H. polymorpha*. Instead we provide evidence that a low pH alone (below pH 5.5) is sufficient to reduce the lifespan. In *S. cerevisiae* the cytosolic pH is kept around 7.0 when cells are grown on glucose even when the external pH is as low as 3.0 26. However, when glucose is depleted a low extracellular pH promotes intracellular acidification to the minimum values of 5.0 - 5.5 27,28,29,30. This process can be stimulated by the presence of low molecular weak organic acids like acetic acid 21,28. As lowering the pH of the medium from 6.5 to 3.5 upon exit from the growth phase decreased the lifespan of cells grown on 0.2% glycerol in a similar way as transfer to new medium / buffer with 2 distinct pH values (Fig. 3B-D), compounds secreted during growth are unlikely to influence the CLS. Instead, we cannot rule out that compounds acting like weak organic acids would be released from dead / lysed cells after the growth phase to accelerate cytosol acidification in the remaining cells.

A weaker impact of buffering on cells grown on 0.6% glucose in comparison to cells grown on glycerol correlates with their elevated resistance to acetic acid (Fig. 4D). It is possible that in cultures containing 0.6% glucose low concentrations of acetic acid, produced as a consequence of the overflow metabolism 19, trigger acid adaptation and cross protection against the effect of low pH later on in the stationary phase. Remarkably, low pH pre-treatment of *S. cerevisiae* cells elevates their resistance to subsequent treatment with acetic acid 31. Such hormetic adaptation and higher initial resistance of 0.6% glucose grown cells to the impact of low pH would explain the limited lifespan extension upon placing these cells into spent medium from cultures containing initially 0.2% glucose (Fig. 2D) and the lower impact of medium buffering on the lifespan of glucose containing cultures (Fig. 4C). We speculate that the adaptation process requires the presence of a weak organic acid (likely acetic acid) in the growth phase and a low pH of the medium. Such adaptation effect could also explain the differences in lifespan observed initially in cultures containing a low and a high concentration of ethanol (which can be converted into acetic acid) and methanol (where formic acid is produced) 32. Consequently, such adaptation may be weak or not occurring in cells grown on 0.4% glycerol rendering these cells more fragile to subsequent exposure to low pH.

The intracellular pH is a parameter that affects a whole range of cellular functions 33,34. The process of cytosolic pH maintenance and weak acid extrusion is energy demanding. In yeast, intracellular acidification activates the accumulation of cAMP 35,36,37 followed by activation of protein kinase A (PKA) targets. Through mobilization of trehalose and glycogen this process can help the cells to overcome ATP shortage 38,39,40. However, low intracellular pH-mediated increase in Ras signalling could also promote chronological ageing via induction of replication stress 22,23. Remarkably, cultures grown on 0.6% glucose also display less budded cells than cultures grown on 0.4% glycerol which is accompanied by less impact of low medium pH on the lifespan of these cells. A stronger arrest in G0/G1 phase after exit from the growth phase observed in cultures containing 0.6% glucose, possibly resulting from the presence of acetic acid in the cultures during the growth phase, could be beneficial in counteracting the induction of growth signalling by the low pH. Consequently, such adaptation is not occurring in the cells grown on high concentration of glycerol, thus these cells are more fragile to the impact of low pH. The fact that a high budding index of the 0.4% glycerol grown cultures is not a problem in buffered medium suggests that the arrest in G0/G1 phase is important for survival only in medium with a low pH. Consequently, when a low concentration of glucose (0.2%) or glycerol (0.1%) is used, the acidification is minor (Fig. 2F) and cells are simply not exposed to low pH.

Altogether our data indicate that the pH of the medium is an important factor determining* H. polymorpha* chronological lifespan. Similarly, the pH was recently shown to affect the chronological senescence in cultured mammalian cells 41,42 suggesting that indeed the mechanism of cellular response to the acidification could be conserved.

The actual effect of dietary restriction strongly depends on the organism and environmental conditions 3. We have shown that acidification impacts the effect of carbon source concentration on lifespan in a carbon source dependent manner. Upon buffering, the chronological lifespan invariably increases with increasing glycerol and glucose concentrations. The differences in lifespan between high and low concentrations of carbon source are mediated by a combination of extracellular and intracellular factors. The actual effect of DR in *S. cerevisiae* depends not only on carbon source concentration, but also on the nutrient composition of the initial medium 2. This yeast also secretes a variety of compounds to the medium 8,9, with further impact on chronological lifespan. Shifting the cells to buffer or water should rule out the impact of extracellular factors on the lifespan of *H. polymorpha*, like previously demonstrated in *S. cerevisiae* 7,43.

In *S. cerevisiae* and other yeast species DR is routinely obtained by reduction of carbon source concentrations from 1%, 2% or higher to 0.5% or less 2,25,44. When *H. polymorpha* cells are grown on 1 or 2% glycerol the CLS is enhanced in comparison to cultures grown on 0.5% glycerol (Fig. S2C). Growth of cells in 1 or 2% glucose resulted in a similar CLS as obtained with 0.5% glucose (Fig. S2D). This data indicate that also at the carbon source concentrations generally used for *S. cerevisiae*, we also see no positive effect of reducing the carbon source concentration on CLS.

Summarizing, our data demonstrate that decreasing the carbon source concentrations in yeast cultures is not a general intervention that leads to an increase in lifespan.

## MATERIALS AND METHODS

### Strains and growth conditions

A wild-type prototrophic strain was obtained by complementation of *H. polymorpha* NCYC495 leu1.1 45 by multicopy integration of pHIPX7 46, containing *S. cerevisiae* *LEU2* gene under its own promoter in the *H. polymorpha* *TEF1* promoter region. Cells were grown in mineral medium 47 containing the indicated carbon sources and 0.25% methylamine as nitrogen source unless stated otherwise. Where indicated, the cells were grown on media containing 0.25% ammonium sulphate or 0.2% urea as nitrogen source. 6 mM K_2_SO_4_ was added when methylamine or urea were used as nitrogen sources. In all experiments the cells were intensively precultivated in MM containing 0.25% glucose and 0.25% ammonium sulphate. When the OD_600nm_ of the precultures reached 1.5-2.0, cells were diluted to OD_600nm_ = 0.1 in the final medium. Culturing was performed in flasks closed with a cotton plug at a medium to flask volume ratio of 1:5, at 37^o^C and with shaking at 200 rpm. When pH control was needed, cells were grown in batch fermenters (culture volume 1 L) in a 2 L fermenter (Applikon, The Netherlands) at 37^o^C, 300 rpm stirring and aeration rate of 0.4 L/min. pH was controlled by the addition of 1 M NaOH.

### Medium swap and buffering experiments

In the spent medium swap experiments cells were spun down for 5 min at 3000 g at 37^o^C, spent medium was collected and clarified by another centrifugation step for 5 min at 3000 g, 37^o^C. The cells were washed once with warm (37^o^C) sterile water and resuspended in the desired spent medium. Cells were similarly transferred to 25 mM phosphate buffer pH 3.5 or 6.5, 50 mM MES buffer pH 3.5 or 6.5 or fresh MM supplemented with 0.25% methylamine.

In buffering experiments spent medium from part of the culture was clarified by centrifugation and the pH was adjusted by addition of 1 M NaOH. Cells from equal volumes of the cultures were recovered by centrifugation and resuspended in filtered and pre-warmed medium with adjusted pH. The same approach was used for buffered spent medium swap experiments.

### Chronological lifespan measurements

The viability of the cultures was assessed essentially as described before 18. Briefly, the number of cells per ml was measured using a CASY Model TT (Roche) and 500 cells were plated on YPD agar plates (1% yeast extract, 1% peptone, 1% glucose, 2% agar) or where indicated YP-glycerol plates (1% yeast extract, 1% peptone, 1% glycerol, 2% agar). After 36-48 hours of incubation at 37^o^C the plates were photographed and colonies were counted using an ImageJ plugin. The number of colonies obtained at the first time point was set as 100%.

### Glycerol, glucose and acetate measurements

Glycerol, glucose and acetate were assayed in clarified medium collected at different time points. For acetate determination the pH of clarified media was adjusted to 7.0 by the addition of NaOH before analysis. Glycerol concentrations were assayed with a Glycerol GK Assay Kit (Megazyme, Ireland), glucose with a D-Glucose HK assay kit (Megazyme, Ireland). Acetate was measured with an Acetic acid assay kit (Acetate kinase analyser format, Megazyme, Ireland). All measurements were performed according to the manufacturer’s protocols.

### Acetic acid resistance and flow cytometry

Cells grown for 20 hours were harvested and resuspended in 50 mM potassium phosphate buffer pH 3.0 and treated with different concentrations of acetic acid (at OD_600__nm _= 0.7) in a 96 well microtiter plate for 1 h at 37^o^C with shaking (900 rpm). The cells were washed once with 50 mM potassium phosphate buffer pH 7.0 and stained for 10 min with 10 µg/ml propidium iodide in the same buffer. After subsequent washing, the fluorescence of 10000 cells was analysed using a FACS Aria II Cell sorter (BD Biosciences) using a 488 nm laser, a 550 nm long pass mirror and a 575/25 nm band-pass filter. Data were recorded and analysed using FACSDiva software (ver. 6.1.2). Stained non-treated and boiled cells were used to set the gates.

### Analysis of budding index

The number of budding cells was determined in cultures grown for 20 hours. Bright field mosaic images were made using a Zeiss Observer Z1 microscope. The number of cells containing a not separated bud was counted manually in at least 500 cells per culture.

## SUPPLEMENTAL MATERIAL

Click here for supplemental data file.

All supplemental data for this article are also available online at http://microbialcell.com/researcharticles/at-neutral-ph-the-chronological-lifespan-of-hansenula-polymorpha-increases-upon-enhancing-the-carbon-source-concentrations/.
