# Supplementary material for: At neutral pH the chronological lifespan of Hansenula polymorpha increases upon enhancing the carbon source concentrations
Source: Microb Cell. 2014 May 20;1(6):189–202. doi: 10.15698/mic2014.06.149 (PMC5354561; doi:10.15698/mic2014.06.149)
Supplement: Supplementary file 1 [file mic-01-189-s01.pdf]

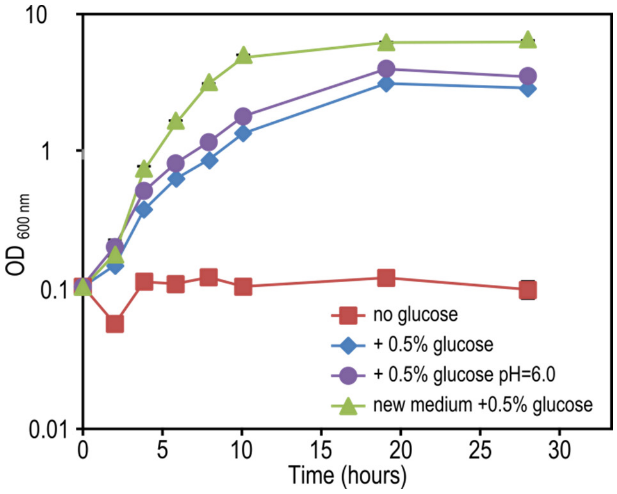

Figure S1 Growth of *H. polymorpha* in fresh and spent medium. *H. polymorpha* cells were grown in mineral medium containing 0.8% glucose and 0.25% methylamine. After 24 hours of growth, the spent medium was recovered by spinning down the cells and subsequent filtration of the medium. Exponentially growing cells (washed in water) were added to the spent medium (no glucose), spent medium with 0.5% glucose (+ 0.5% glucose) or to medium with pH adjusted to 6.0 using concentrated sodium hydroxide (+0.5% glucose pH=6.0). The same cells were shifted to fresh mineral medium (new medium +0.5% glucose). Data represent mean OD 600nm ± SD (n=3).

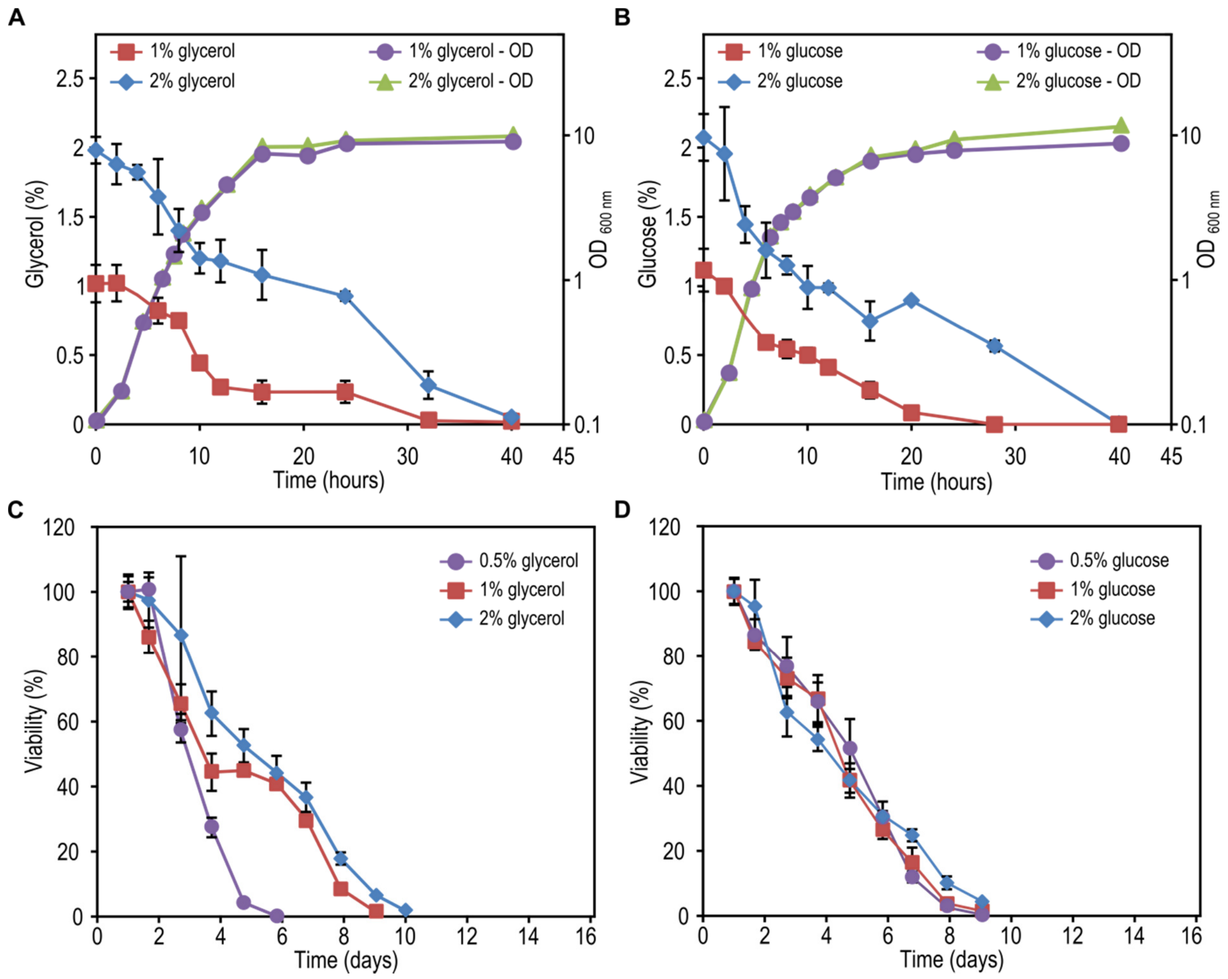

Figure S2 Chronological lifespan of *H. polymorpha* cells in medium containing 0.5%, 1% and 2% of glycerol or glucose. Growth and carbon source concentration in the medium in cultures containing 1% and 2% glycerol (**A**) or 1% and 2% glucose (**B**) and 0.25% methylamine. Chronological lifespan of cells grown 0.5%, 1% and 2% glycerol (**C**) or 0.5%, 1% and 2% glucose (**D**) Data represent mean concentration ± SD and mean OD $_{600nm}$ ± SD. Chronological ageing experiments represent mean viability ± SD (n=3).

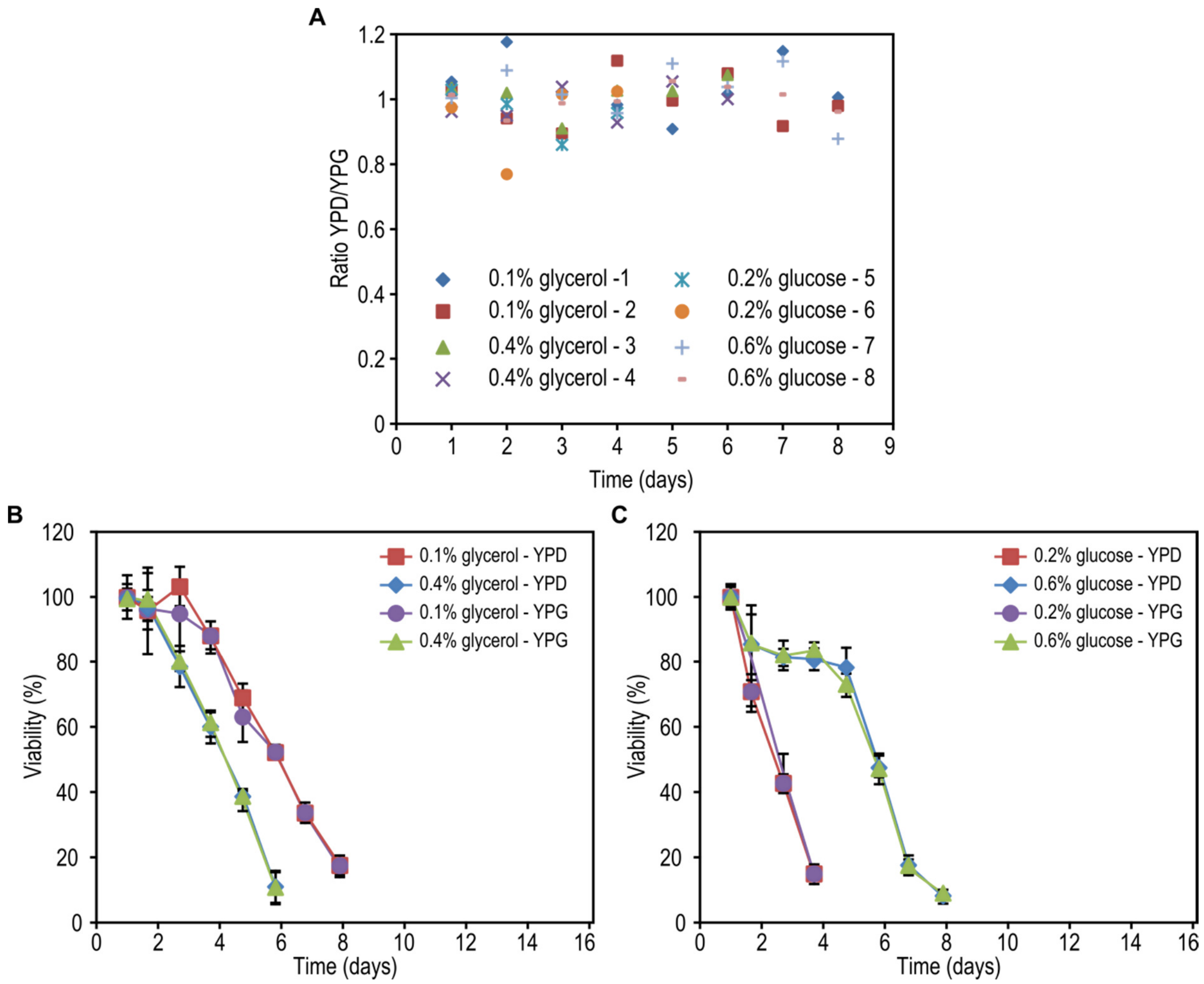

Figure S3 Chronological lifespan experiments using YPD or YP-glycerol (YPG) plates to determine the colony forming units. Cells were grown in mineral medium containing 0.1% or 0.4% glycerol and 0.2% or 0.6% glucose. The ratios of colonies obtained after plating on YPD and YPG for individual cultures (1-8) were plotted in time (**A**). The YPD/YPG ratios were always close to 1, independent of the time point indicating that the carbon source in the plates does not affect the obtained results. Chronological lifespan of the cells grown on 0.1% or 0.4% glycerol (**B**) and 0.2% or 0.6% glucose (**C**) using either YPD or YPG plates for assessing the viability. Data represent mean viability ± SD (n=2).
